# Anogenital scent-marking signals fertility in a captive female Alaotran gentle lemur

**DOI:** 10.3389/fvets.2022.940707

**Published:** 2022-07-28

**Authors:** Sara Fontani, Stefano S. K. Kaburu, Giovanna Marliani, Pier Attilio Accorsi, Stefano Vaglio

**Affiliations:** ^1^Animal Behaviour and Wildlife Conservation Group, School of Sciences, University of Wolverhampton, Wolverhampton, United Kingdom; ^2^Animal Behaviour and Wildlife Conservation Group, School of Medicine, University of Wolverhampton, Wolverhampton, United Kingdom; ^3^Dipartimento di Scienze Mediche Veterinarie, Universitá di Bologna, Bologna, Italy; ^4^Behavioural, Ecology and Evolution Research Centre, Durham University, Durham, United Kingdom

**Keywords:** captive breeding, chemical signalling, faecal endocrinology, *Hapalemur alaotrensis*, reproductive biology

## Abstract

The Lake Alaotra gentle lemur (*Hapalemur alaotrensis*) is one of the 25 most endangered primates in the world and shows low success rate in captive breeding programmes. It is therefore vital to further understand its reproductive biology. We studied a captive troop consisting of five individuals hosted at Jersey Zoo during breeding and non-breeding periods over 1 year. We collected behavioural data (*n* = 318 h) using all occurrence of some behaviours and *ad libitum* sampling methods, as well as faecal (*n* = 54) and anogenital scent (*n* = 35) samples of the breeding female. We measured sex hormone levels using enzyme immunoassay technique and investigated the volatile component of odour signals using solid-phase microextraction and gas chromatography-mass spectrometry. We observed sexual and aggressive behaviours occasionally during the breeding period. Our regression analysis showed that only period significantly predicted rates of female anogenital scent-marking, whereby the female performed anogenital scent-marking more frequently during the breeding rather than the non-breeding period. In contrast, female hormone levels did not significantly explain variation in rates of neither male nor female olfactory, sexual and affiliative behaviours, suggesting that individuals' behaviour alone is not an effective indicator of the ovulation window. The volatile chemical profile of anogenital odour secretions changed over the study, with four compounds distinguishing the fertile window during the breeding period. In conclusion, our findings suggest that anogenital scent-marking may signal the reproductive status of captive female gentle lemurs.

## Introduction

Of 504 primate species recognised today worldwide, almost half are classified as endangered or critically endangered in the wild—primarily due to human impact; thus, raising global scientific and public awareness of the plight of the world's primates is now vital (1, 2). The most important actions needed for ensuring the survival of these irreplaceable species are conservation, research, public education and outreach, wherein zoos play a major role (3, 4).

Among captive animals, zoo populations are unique as they are usually managed to educate the public regarding wildlife and their habitats, and to preserve endangered species through captive breeding and reintroduction programmes (5–7). In this context, the maintenance of genetic variation, and thus high survival rate in case of reintroduction, is imperative (8, 9). However, captive populations, potentially serving as buffers against extinction, are experiencing problems that impair them from being viable for reintroduction into the wild. Particularly, zoo populations face reproductive challenges which may prevent them from serving as viable “reserve populations” (10).

To maintain captive healthy populations, modern zoos take part in conservation breeding programmes (5, 6). Moreover, as reproductive success is linked to how closely captive environmental conditions mirror those that primates would be experiencing in the wild (10), zoos also use environmental enrichments to manage captive populations [including scent enrichment programmes (e.g., (11))]. Environmental enrichments and conservation breeding programmes are linked, as enrichment is a dynamic process that changes an animal's environment, increasing its behavioural choices and prompting a wider range of natural and species-specific behaviours and abilities (12).

Among all primate species, lemurs are the most endangered. Almost a third of the 107 species living in Madagascar are listed as critically endangered, while 98% of them are threatened by extinction (13). Moreover, several captive lemur populations are struggling, in terms of abundance and demographic trend, and currently would not support reintroduction into the wild (10).

The Lake Alaotra gentle lemur (*Hapalemur alaotrensis*) is one of the five lemur species included in the 2018–2020 list of The World's 25 Most Endangered Primates (14). It is the only lemur species living exclusively in a wetland and its limited geographical range, combined with increasing habitat degradation and hunting pressure, have brought this species to the brink of extinction (15, 16). With a population continuously declining, it is estimated that only around 2,500 individuals remain in the wild (16).

Given the danger that it faces, the gentle lemur is now targeted by several conservation initiatives (16), such as the European Association of Zoos and Aquaria (EAZA)'s *Ex-situ* Programme (EEP), which aims to maintain captive healthy populations and reduce the loss of genetic variation, which can be rapid in small captive populations (8, 17, 18), through breeding management recommendations. Nevertheless, gentle lemurs are currently showing a low success rate in captive breeding across EAZA institutions (19). In this context, it is becoming increasingly important to fully understand their reproductive biology in order to try and affect positively their conservation status (20).

It is now widely acknowledged that olfaction plays an important role in socio-sexual communication and reproductive biology in non-human primates [e.g. (21–28)]. Although little is known about gentle lemurs, it is broadly established that lemurs rely heavily on olfaction [reviewed by (29)], with semiochemicals being important for territorial marking, social communication, kin recognition and mate choice (30–34). Among primates, lemurs show the utmost variability and specialization in chemosignalling, with a great diversity in design, delivery and perception of chemical signals. They actively scent mark, have a functional vomeronasal organ, which is very sensitive to chemical messages, and investigate scents *via* olfactory and gustatory means [reviewed by (35)]. Scent signals can be released through specialized glands, urine and faeces, sweat and skin (36); variation in scent mixing, delivery and multimodality alters signal longevity and intended receivers [reviewed by (37)]. Scent release can also vary both quantitatively (frequency of scent marking and amount of scent deposited) and qualitatively (spatial and seasonal distribution and substrate marked) [reviewed by (29)]. Moreover, signs of marking can be persistent in the environment [e.g., faecal or urinary latrines (38, 39)]. The information released with chemical signals changes the behaviour or perception of the receiver in a way that generally benefits both sender and receiver (40), typically in the service of mutually profitable, socio-reproductive functions (29). In several lemur species, female olfactory signals can advertise their reproductive status, varying between the breeding and non-breeding seasons (30, 41, 42). Thus, chemicals have also great potential as tools to trigger olfactory and sexual behaviours in lemurs (24, 36, 43), which is important in critically endangered species.

In this study, we combined behavioural observations of olfactory and sexual behaviours with the chemical investigation of anogenital odour secretions and the timing of fertility, obtained *via* measurement of sex hormone levels, in a zoo-housed gentle lemur. In particular, we aimed to:

- Detect the female fertile window using faecal progesterone and oestradiol levels.
- Examine the relationship between female and male behavioural patterns (focusing on olfactory and sexual behaviours) and female sexual cycle stages.
- Identify the key compounds that convey information about female fertility.

## Materials and methods

### Study subjects and periods

We studied a captive family group of gentle lemurs (*n* = 5), consisting of a breeding pair and their offspring (Table 1), hosted at Jersey Zoo—formerly Durrell Wildlife Park (Channel Islands).

**Table 1:** Jersey Zoo study group.

**Name**	**Sex**	**Age at study start**	**Age at study end**
*Miora*	Female	7 years, 2 weeks	7 years, 6 months
*Nova*	Male	13 years, 3 months, 3 weeks	13 years, 9 months
*Ririnina*	Male	1 year, 7 months, 2 weeks	2 years, 3 weeks
*Twin 1*	Unknown	1 month, 1 week	6 months, 3 weeks
*Twin 2*	Unknown	1 month, 1 week	6 months, 3 weeks

Female gentle lemurs are able to reproduce at 2 years, whereas males are sexually mature at 3 years [wild: (44), captivity: (45)]. The average lifespan in captivity is 17.1 years for females and 12.8 years for males (46). Gentle lemurs are seasonally polyoestrous and mating occurs over 1 day per sexual cycle (47). They usually deliver offspring once per year (47) after an average gestation period of 145 days (45), with a high rate of twinning (44). In Madagascar the mating season occurs during the dry season [i.e., between April and September (44)], while there is no defined breeding season in captivity (45). We estimated the breeding and non-breeding periods on the basis of the last parturition of the female and the length of the weaning period (48, 49).

Sampling (breeding and non-breeding) periods, including behavioural observations as well as collected faecal and odour samples, are detailed in Table 2.

**Table 2:** Overview of the data collection.

**Sampling periods**	**Non-breeding period**	**Breeding period**
Dates	02/08/2021–14/09/2021	01/12/2021–11/01/2022
Hours of behavioural observations	204	114
Faecal samples	30	24
Anogenital odour samples	11 (2)	24 (4)

*Control samples are in brackets*.

### Data collection

#### Faecal hormone sampling and measurement

We collected faecal samples (*n* = 54) from the breeding female every morning during the behavioural observations, when defecation was observed, and the identity of the animal was certain. We immediately stored the samples in a −20°C freezer at the zoo and then transferred them, using a freezer box with ice packs to avoid any risk of defrosting, to the Rosalind Franklin Science Centre, University of Wolverhampton, for laboratory analyses.

##### Hormone analyses

We lyophilized the faecal samples for 72 h using a freeze-drying machine (Christ^®^, Beta 1–8 LSC plus, Osterode am Harz, Germany), then we pulverized and sieved them to separate the faecal residue from the fibrous material. The extraction methods were based on those detailed in Maréchal et al. (50). Briefly, we extracted 0.05–0.1 g of faecal powder in 3 ml of 80% methanol in a 15 ml plastic tube. Then, after vortexing for 15 min using a multi-tube vortexer (Grant Instruments^®^, Multi-Vortexer V-32, Cambridge, UK) and centrifugation for 20 min at 3,266 xg, we immediately stored the supernatant at −20°C. We excluded a faecal sample from the analysis as it was degraded.

##### Enzyme immunoassays

We measured progesterone metabolites and 17β-Estradiol levels using ELISA kits (DetectX Progesterone metabolites K068-H5 and DetectX 17β-Estradiol K030-H5, Arbor Assays^®^, USA) following the protocol detailed in (51). We diluted the samples 1:10 with the assay buffer and run all assays according to kit instructions. We assayed all faecal samples and standards in duplicate. We analysed assay data utilizing a 4-parameter logistic (4PL) fitting programme (MyAssays^®^, Brighton, UK). Concentrations were expressed as pg/mg.

Mean intra-assay coefficients of variation of four samples, tested with eight replicates within a single assay plate, was 10.2% for progesterone and 7.6% for oestradiol. Mean inter-assay coefficients of variation of four quality control samples, measured in duplicate across three assay plates, was 12.3% for progesterone and 8% for oestradiol.

##### Analytical validation

We conducted a parallelism test between serial dilutions of two samples (A and B) and the standard curves of each kit to validate the enzyme immunoassays (52). We performed a Pearson correlation test to assess the strength of the association between the slopes of the standard curves and that of the diluted samples [Progesterone-Sample A: *r*_3_ = 0.99, *p* = 0.001; Progesterone-Sample B: *r*_3_ = 0.99, *p* = 0.001; Oestradiol-Sample A: *r*_3_ = 0.96, *p* = 0.007; Oestradiol-Sample B: *r*_3_ = 0.97, *p* = 0.006].

##### Interpretation of faecal hormone profiles and definition of the oestrus cycle

We used the patterns of faecal progesterone metabolites and 17β-oestradiol levels to determine the occurrence of ovulatory windows and the timing of fertility in the breeding female [we considered the time lag between steroid secretion and excretion in faeces (53)]. We estimated an ovulatory window when oestradiol increased along with a progesterone decrease followed by a constant progesterone rise for at least 5 days. This approach was used to define the ovulatory phase of the ovarian cycle and has previously been shown to be valid for assessing the timing of ovulation in other lemur species (54–56).

#### Behavioural observations

We collected behavioural data from early mornings to early afternoons (non-breeding period: 6 h/day; breeding period: 4 h/day), 5 days per week (non-breeding period: Mondays to Fridays; breeding period: Sundays to Tuesdays and Thursdays to Fridays), during two study periods (non-breeding period: August/September 2021; breeding period: December 2021/January 2022), for a total of 318 h. We conducted all occurrence and *ad libitum* sampling sessions (56, 57) to collect data on olfactory, sexual, affiliative and aggressive behaviours using an ethogram developed by Errington (58) and then modified using prior studies by other authors (45, 47, 59) (Table 3).

**Table 3:** Ethogram of selected behaviours for the study subjects.

**Behaviours**	**Key**	**Description**
**Olfactory behaviours**		
Brachial scent marking	BSM	Male. Scratch object with lower dentition, then rubbing spot on brachial glands (on arms)
Anogenital scent marking	ASM	Female. Rubs object with genitalia, then sit-rubbing repeatedly whilst depositing urine
Sniff genitals	SG	Male. Place the nose <3 cm from the anogenital area of a conspecific and lick it
**Sexual behaviours**		
Anogenital self grooming	ASG	Grooming of genital area, using fingers or mouth
Follow	FOL	Male approaches female from behind and follows closely (<1 m)
Mating calls	MT	Females produce distinct singly or in series call, while soliciting copulation and during mating
Attempt mounting	AM	Male approaches female, clasps, orients body for copulation; female chatters at and/or cuffs the male, male releases female
Copulation	CO	Male approaches, female crouch, male introduces sperm into the female's reproductive tract
**Affiliative behaviours**		
Grooming	GR	Using fingers or mouth to pick through the coat, removing any foreign bodies from a conspecific
**Aggressive behaviours**		
Intimidation	INT	Animal emits a short vocalization toward a conspecific to warn it not to come closer

#### Odour sampling and investigation

Before data collection, we used positive reinforcement training (60) for 5 days to train the breeding female to allow us to collect anogenital odour secretions.

We collected anogenital odour samples (*n* = 35) every morning before behavioural observations by rubbing 10 times a sterile cotton swab around the wall of the vulva, using steady pressure, as previously described by Vaglio et al. (28). Moreover, we exposed control swabs to the air once a week to identify any compounds that did not derive from the lemurs. We placed all samples and controls into sterile vials (61) and immediately stored them in a −20°C freezer at the zoo. We then transferred the vials to the Rosalind Franklin Science Centre, University of Wolverhampton, using a freezer box with ice packs to avoid any risk of defrosting, for laboratory analyses.

##### Odour analyses

We investigated the volatile component of odour signals using solid-phase microextraction (SPME) and gas chromatography-mass spectrometry (GC-MS) techniques, as previously described by (61).

Briefly, we introduced a 65 μm polydimethylsiloxane/ divinylbenzene SPME syringe needle through the vial septum and exposed the fibre to the headspace above the sample in the vial for 15 min at 40°C. We analysed the adsorbed volatile analytes of all samples using a 5975C mass spectrometer (Agilent Technologies) EI, 70 eV, coupled directly to a 7890B gas chromatograph (Agilent Technologies) equipped with a fused silica HP5-MS UI capillary column (Agilent Technologies) 30 m × 0.25 mm crossbonded 5%-phenyl-95% dimethylpolysiloxane, film thickness 0.25 μm. We maintained the injector and transfer line temperatures at 270°C and 280°C, respectively.

We made injections in splitless mode (purge valve opened after 1 min) with a constant flow of helium carrier gas of 1 ml min −1. We started the oven temperature program at 45°C for 2 min, then raised it by 4°C min −1 to 170°C, and finally by 20°C min −1 to 300°C 40.

We assessed possible environmental contamination *via* blank analyses using an empty 10 ml vial (Supelco) and control swabs following the same procedure as for the samples and conditioned the fibre at 260°C pre-injection for 5 min and 260°C post-injection for 20 min to avoid any possible carry-over effects.

We standardized peak retention times using retention time locking to alpha pinene. We tentatively identified eluted compounds by comparing the experimental spectra with those of the mass-spectral library in ChemStation (Agilent Technologies) and NIST Database (National Institute of Standards and Technology), version MSD F.01.01.2317 (Agilent Technologies). We accepted a putative identification if the minimum matching factor was higher than 90%. To minimize the chance of misidentification and when more than one compound was a good match for the same GC peak, we considered the chromatographic retention time and compared it with those reported in the literature for the same chromatographic column type. We created a data matrix using the peak area relative to each identified compound using the integrated signal of the deconvoluted total ion current (TIC).

We analysed all samples in a short period of time to minimize inter-assay variability. We overlaid chemical profiles from control swabs on animal chemical profiles to identify compounds that did not derive from the animals and removed these from the swab results.

### Data analysis

In order to assess whether male and female behaviours were significantly affected by female oestradiol levels and/or period (breeding vs. non-breeding), we conducted a series of linear regressions. All models included both female oestradiol concentrations and period as predictors, while, in separate models, we included as dependent variables female anogenital behaviour as well as male follow, sniffing, sexual inspection and grooming behaviours. We applied model validation on all regression models to verify the underlying assumptions, by checking whether residuals were normally distributed and by plotting residuals versus fitted values to assess homogeneity of variance. None of the assumptions were violated. We performed statistical analyses using the “lm” function implemented in R Studio software (version 4.1.1) (62).

### Ethical statement

We conducted this study using non-invasive techniques, in compliance with the Directive 2010/63/EU and Decision 2014/11/EU. The study protocol was approved by the Life Sciences Ethics Committee of the University of Wolverhampton (UK) and the Ethics Committee of the Jersey Zoo—Durrell Wildlife Conservation Trust (Channel Islands).

## Results

### Faecal hormones

**Figure 1:**
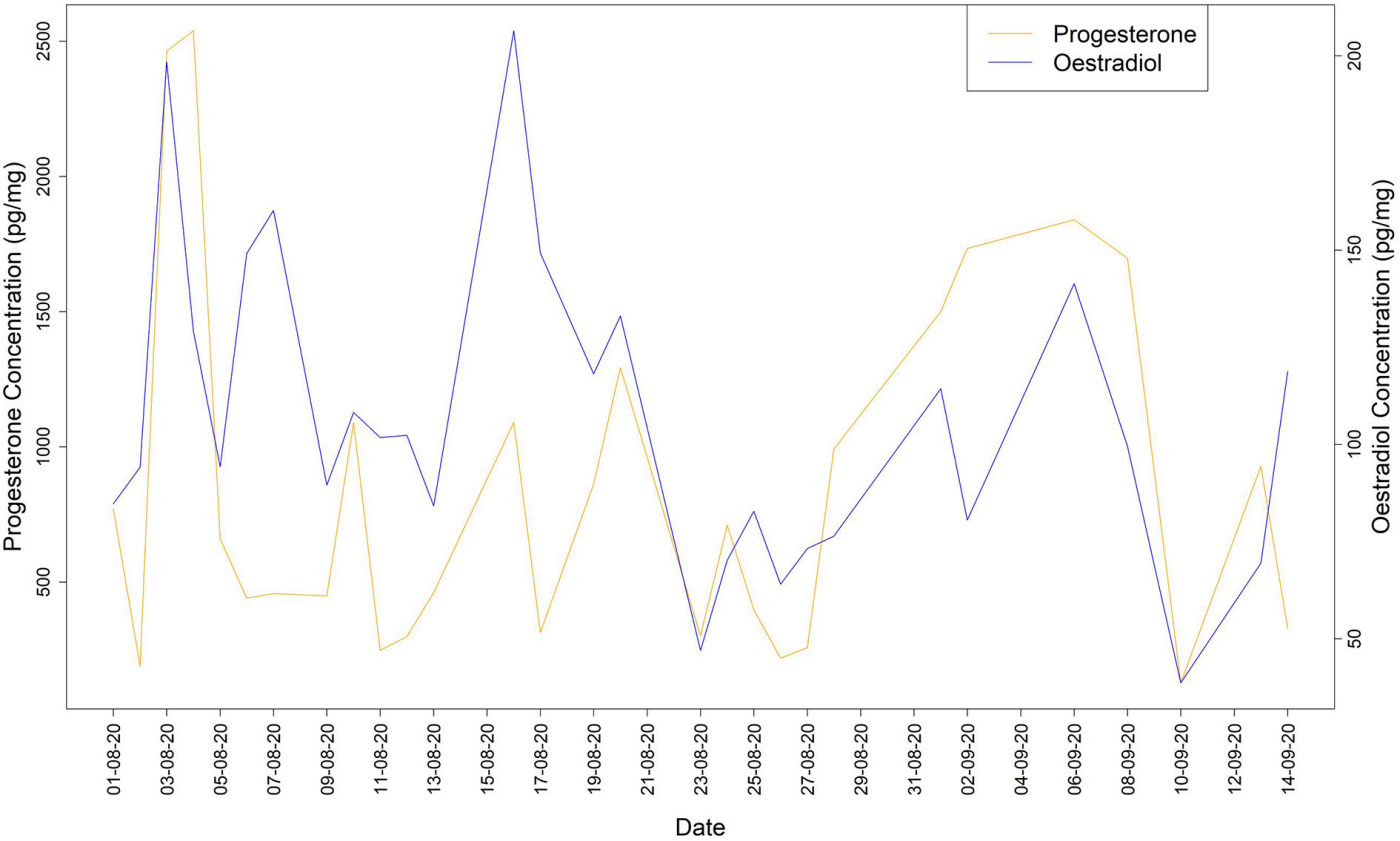
Oestradiol and progesterone concentrations during the non-breeding period.

During the non-breeding period, the profiles of both sex hormones (progesterone and oestradiol) showed a synchronized pattern with daily variations, which did not indicate any ovulation (Figure 1).

**Figure 2:**
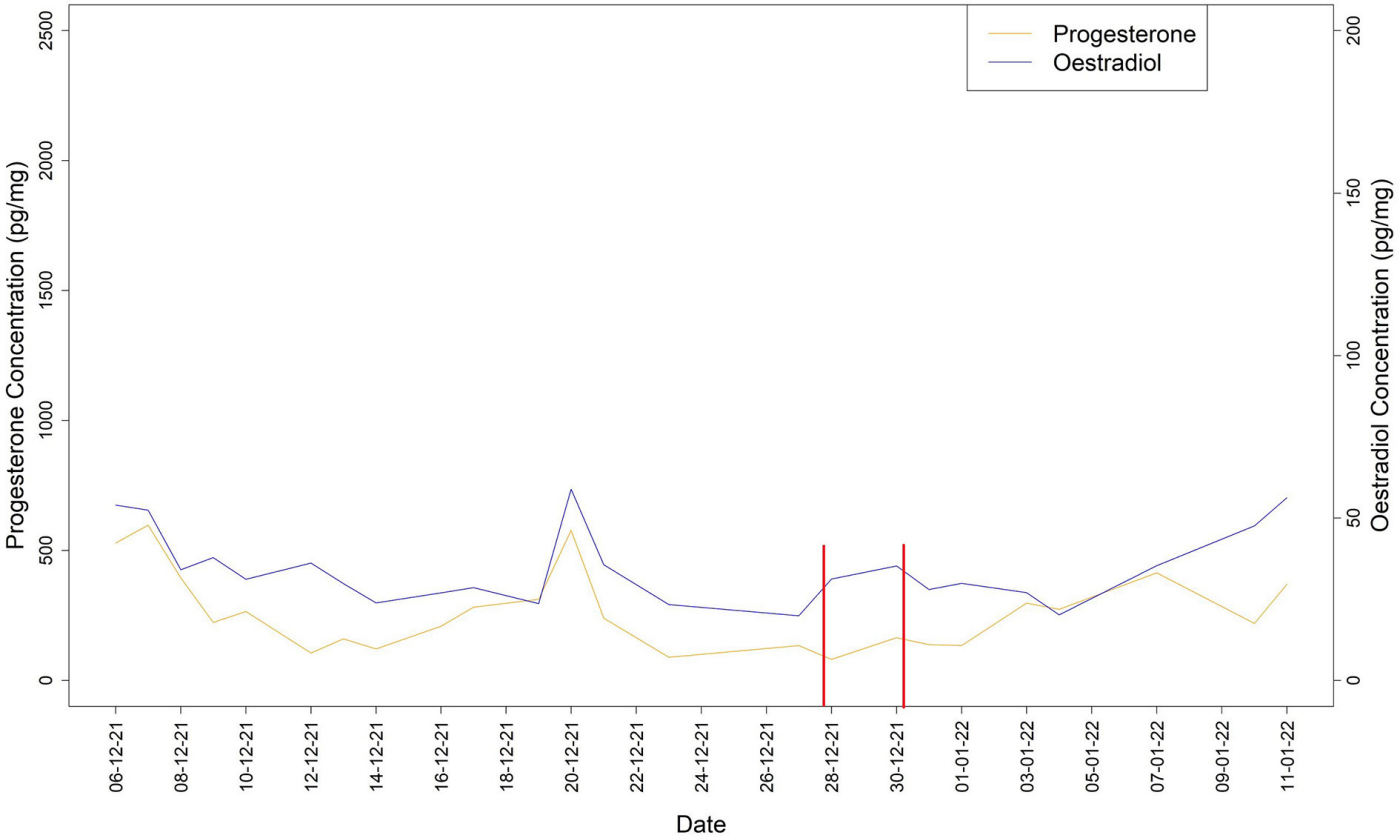
Oestradiol and progesterone concentrations during the breeding period. Vertical bars denote the ovulation window.

During the breeding period an increase in oestradiol concentration, accompanied by a decrease of progesterone, occurred both on December 11th and 29th. In both cases oestradiol peaks were followed by a constant increase of progesterone levels that reached a peak after 8 days (Figure 2).

### Olfactory, sexual and affiliative behaviours

The regression analyses testing the effect of female oestradiol concentrations and period on male and female behaviours showed that the only model that was significant was the one that included rates of female anogenital scent-marking as dependent variable [*F*_2, 34_ = 8.98, *p* = 0.020, *R*^2^ = 0.16]. This model showed a significant effect of period (but not oestradiol levels) on rates of female anogenital scent-marking (Table 4), with higher frequencies of anogenital scent-marking occurring during breeding (0.86 N/h.) than non-breeding period (0.05 N/h.). Similarly, we found a trend in the model that included rates of male genital sniffing followed by scent-marking [*F*_2, 34_ = 3.11, *p* = 0.06, *R*^2^ = 0.10], with a significant effect, again, of period on male behaviour (Table 4): males performed more frequent genital sniffing followed by scent-marking during the breeding (1.98 N/h) than non-breeding period (0.97 N/h). None of the other regression models was significant (Table 4). We observed male and female anogenital self-grooming only during the breeding period, twice 2 days before copulation occurred. We observed intimidation behaviours from the adult male towards the subadult male three times 2 days before copulation and once the day of copulation. We observed attempted mounts on December 14th, 21st, and 24th, while copulation occurred five times with different durations, over an hour, on December 30th. We did not document any mating call. We did not observe any sexual behaviour after copulation occurred on December 30th. See Table 5 for a summary of such *ad libitum* observations.

**Table 4:** Summary of the linear regression results testing the effect of female oestradiol concentrations and periods (breeding vs. non-breeding) on female and male behaviours.

**Dependent variable**	**Independent variable**	**Estimate**	**SE**	***T*** **-test**	***P*** **-value**	*R* ^2^
Female anogenital scent marks	Intercept	−0.02	0.44	−0.04	0.970	0.16
	Oestradiol concentrations	0.0006	0.004	0.17	0.868	
	Season	0.85	0.39	2.19	0.036	
Male follow	Intercept	0.10	0.15	0.68	0.505	
	Oestradiol concentrations	0.0008	0.0013	0.65	0.521	−0.003
	Season	0.18	0.14	1.31	0.198	
Male sniff genitals	Intercept	0.81	0.78	1.04	0.305	
	Oestradiol concentrations	0.0002	0.0063	0.04	0.971	0.06
	Season	1.03	0.69	1.50	0.142	
Male sniff genitals followed by scent marks	Intercept	0.57	0.68	0.85	0.403	0.10
	Oestradiol concentrations	0.00	0.01	0.63	0.533	
	Season	1.28	0.60	2.14	0.040	
Grooming	Intercept	0.07	0.24	0.27	0.786	
	Oestradiol concentrations	0.0018	0.0020	0.94	0.356	−0.03
	Season	0.12	0.21	0.56	0.579	

Significant results are indicated in bold.

**Table 5:** Overview of the behavioural observations (NO, Not-Observed).

**Behaviours**	**Non-breeding period**	**Breeding period (Ovulations occurred on December 11 and 29th)**
Mating calls	NO	NO
Anogenital self-grooming	NO	28/12/2021
Male-male intimidation	NO	28/12/2021, 30/12/2021
Attempted mounts	NO	14/12/2021, 21/12/2021, 24/12/2021
Copulation	NO	30/12/2021

### Odour secretions

**Figure 3:**
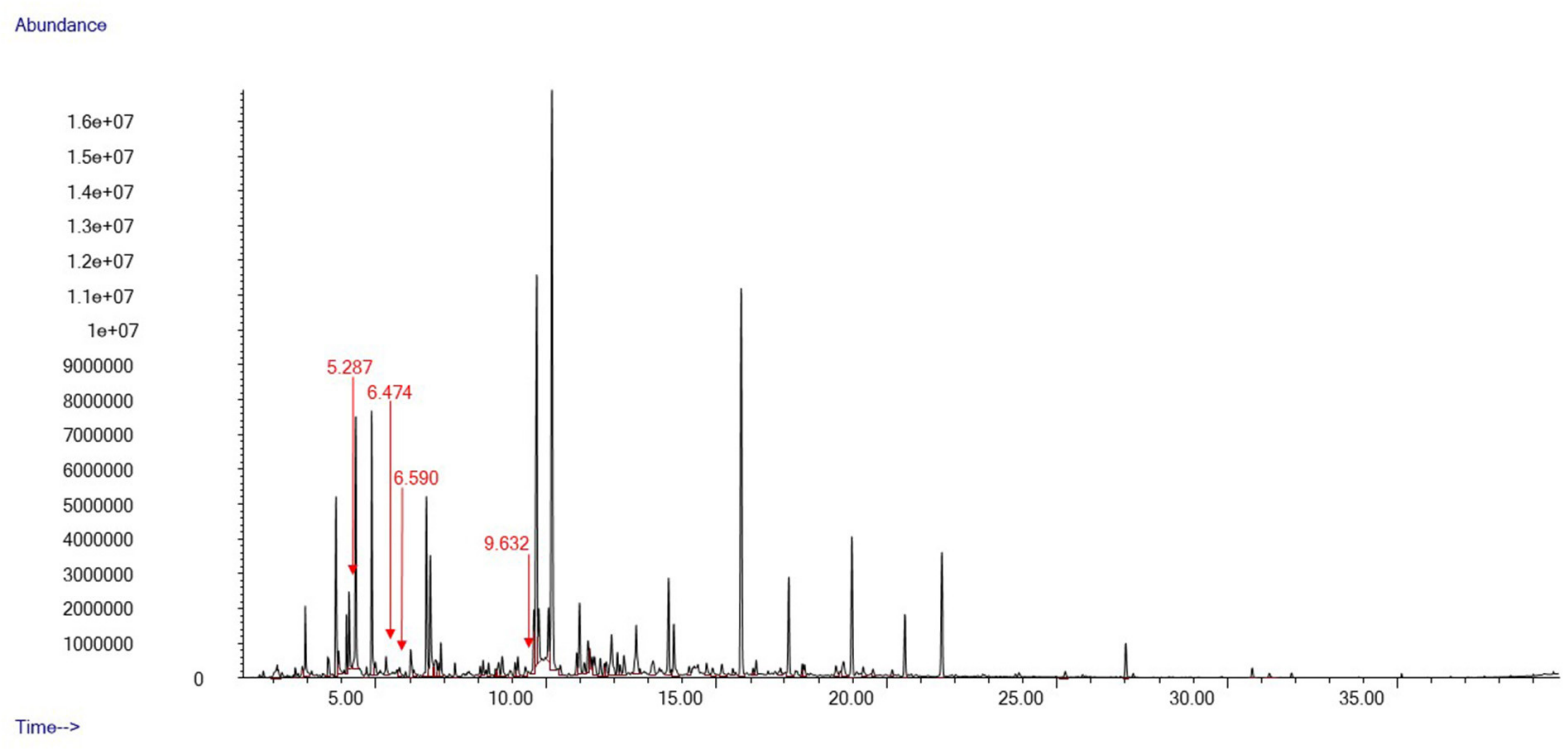
Oestradiol and progesterone concentrations during the breeding period. Vertical bars denote the estimated ovulation window.

**Figure 4:**
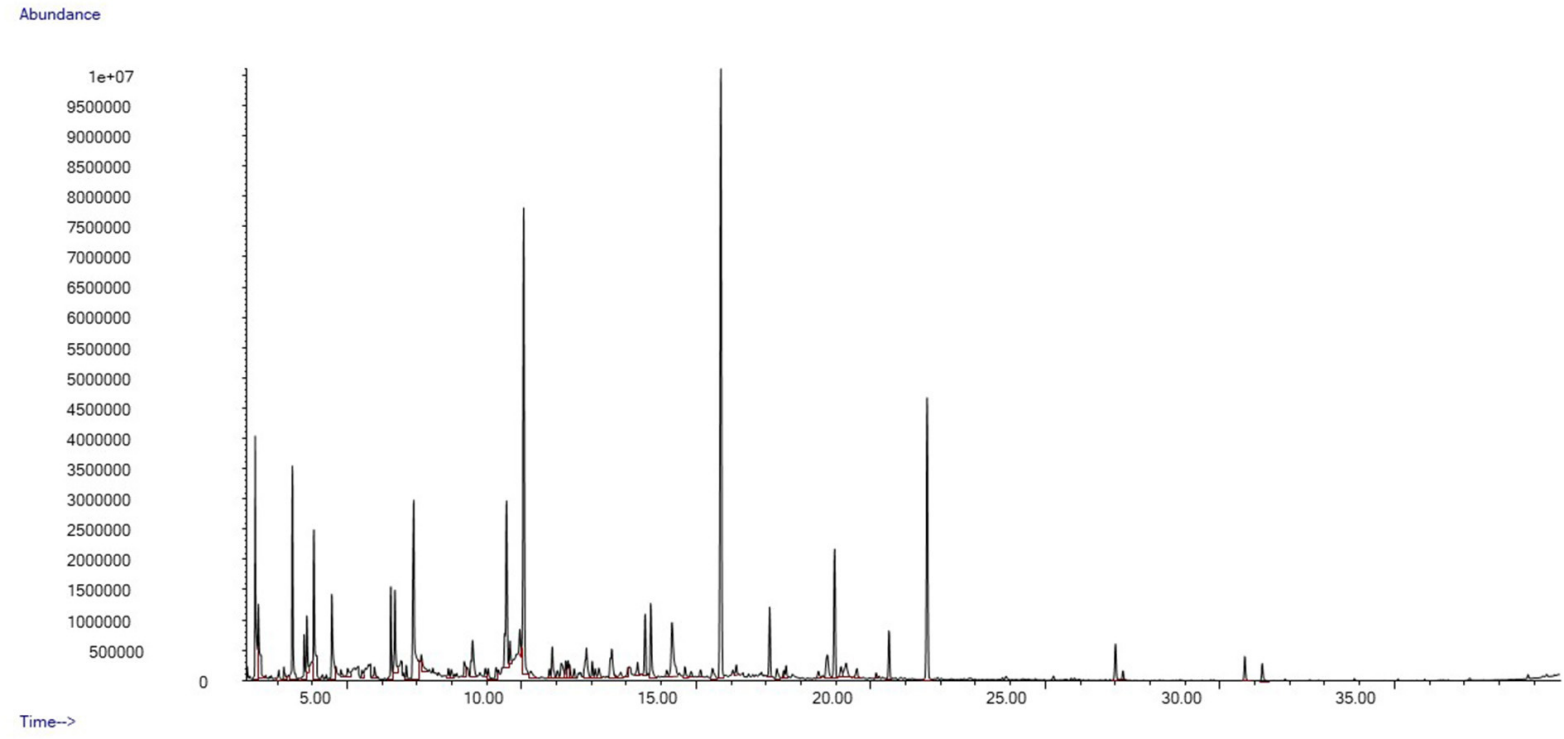
Example chromatogram from female gentle lemur, anogenital odour sample during the non-fertile period.

We identified a total of 78 distinct peaks in 35 swab samples of gentle lemur anogenital secretions that were not present in the control swabs. These compounds included a series of hydrocarbons and fatty alcohols, organic aliphatic acid esters and carboxylic acid, aldehydes, and aliphatic ketones. Typical chromatograms from the fertile and non-fertile periods are shown in Figures 3, 4, respectively.

Particularly, a small pool of compounds was only present in the chemical profiles of anogenital odour samples collected during the second ovulation window (28th–30th December 2021). Three compounds were tentatively identified as 3-Heptanone, 2-Heptanone, and 3-Octanone, whilst one could not be given any tentative identification (Figure 3). These compounds were unique to the second ovulation window (28th–30th December 2021) during the breeding period.

## Discussion and conclusions

Little is known about the chemical changes underlying reproductive quality in non-human primates due to methodological challenges in sampling and quantifying odour profiles (21). In this study we determined the timing of female fertility in a gentle lemur using observations of sexual behaviours and faecal endocrinology (63), and examined the relationship between the reproductive status of the female, anogenital scent, and both female and male behavioural patterns.

Faecal hormones are commonly used to acquire endocrine information on ovarian function in individual female primates (64). We found that faecal hormone trends were different between the study periods (non-breeding vs. breeding). Particularly, during the non-breeding period the profiles of progesterone and oestradiol showed a synchronized pattern, while during the breeding period two potential ovulatory events were estimated. In the context of the breeding period, as observed in other lemur species [e.g., *Lemur catta* (54, 55), *Lemur variegatus* (59), *Eulemur mongoz* (56)], the female oestradiol level increased, reaching a peak the day before mating occurred, alongside a decrease of the progesterone level followed by a slow steady rise until reaching a peak 8 days after ovulation. This is consistent with what has been reported in other lemurs; i.e., oestradiol peak usually occurs on the first day of vaginal oestrus and receptivity usually follows about 1 day after the oestradiol peak, oestradiol levels fall on the day after the oestradiol peak and females become sexually receptive during this period (55, 59). The tentative interval between the two ovulation windows was around 20 days. Although this interval is slightly different in comparison to what has been reported in other lemurs (i.e., *Lemur macaco*: 24–28 days, *Lemur variegatus*: 30–36 days, *Eulemur mongoz*: 26–35 days), a degree of variability both intra- and inter-species has also been suggested by other authors (54, 56, 59).

We also found that the average level of both hormones was higher during the non-breeding than the breeding period. This may be caused by several factors. First of all, the diet, as during the summer (non-breeding period) the daily diet of the study group included large amounts of bamboo; i.e., a plant rich in flavonoids (phytoestrogens) which may impact on female sex hormones. Particularly, it has been found that phytosteroids affect the reproductive hormone levels in both humans and non-human animals (65, 66), while many studies on dietary phytoestrogens highlight the potential effects of total flavonoids in terms of increasing progesterone and oestradiol levels (66). In addition, higher hormone levels over the summer can be due to seasonal variation, as progesterone and oestradiol tend to increase when the photoperiod lengthens (67), and the reproductive history of the female, who had gone into oestrus during previous summer seasons but at the time of this study was lactating over the summer and the presence of a suckling infant may have delayed the onset of ovulation (68).

Since we did not observe any attempted mounts or aggressive behaviours before the first ovulation window (9th–11th December 2021), we suggest that this was a silent ovulation event (i.e., an ovulation not accompanied by perceptible morphological changes in the anogenital area and during which no sexual behaviour occurs). Silent ovulations have been reported in several mammal species, including non-human primates (69, 70). In addition, based on our endocrinological and behavioural data, we estimated an ovulation window occurring on 28th–30th December 2021; as further verification that the female was ovulating, she became pregnant following copulation and gave birth to twins after 142 days of gestation. As in other lemur species [e.g., *Lemur catta* (71), *Eulemur fulvus* (72), *Lemur variegatus* (59)], within each breeding period the gentle lemur's female fertile window would then be restricted to 2 days per oestrus cycle (47).

With regards to the relationship between the reproductive status of the female and both female and male behavioural patterns, we found a significant effect of period (breeding vs. non-breeding) on female anogenital scent-marking and male sexual behaviour (i.e., sniff genital followed by scent-marking) but we did not find any significant effect of female hormones on either female marking behaviour or male olfactory, sexual and affiliative behaviours. These results suggest that male or female behaviours alone cannot be considered as an effective indicator of female fertility in gentle lemurs.

Although it is known that in other lemur species [e.g., *Eulemur fulvus* (73), *Lemur variegatus* (59)] male behavioural responses towards female fertile signals consist of increased frequency of behaviours such as follow, sniff genital and olfactory inspections of scent-marks, and more aggressive interactions between males [e.g., *Microcebus murinus* (74), *Lemur catta* (75), *Propithecus verreauxi* (76)] our findings are not unexpected. In gentle lemurs it is not unusual that activity levels remain mostly unchanged throughout the year (47), while oestrus cycles cannot be detected (45). Moreover, sexual behaviours may be missed or underestimated by the observers due to lemur cathemeral activity rhythm (77, 78). In addition, in some primate species, such as Hanuman langurs [*Semnopithecus entellus* (79)] and rhesus macaques [*Macaca mulatta* (80)], male behaviour seems not correlated with female ovulation.

Female primates use a variety of sexual signals to communicate their fertility to males, such as visual (81) [e.g., sexual swelling (82, 83) and proceptive behaviour (84)], acoustic [e.g., copulation calls (85–87)] and olfactory signals (24, 28, 88, 89). We investigated anogenital odour secretions released by a female gentle lemur and detected a small pool of compounds distinguishing the volatile chemical profile during the fertile period. Particularly, we found four volatile compounds (2-Heptanone, 3-Heptanone, 3-Octanone, and another compound that could not be identified) which were only present during the two-day ovulation window.

2-Heptanone, an aliphatic ketone insoluble in water, has been identified acting as a pheromone in both female and male mice urine (90). In females, it delays puberty in conspecifics (91) and promotes oestrous (92), while in males it has a function of stress indicator for conspecifics (91) and its content increases when the signaller is subjected to stressful events (93). It was also found having a putative semiochemical function in Macleay's marsupial mice (*Antechinus stuartii*), with a difference between intact and castrated males (94); in male brown rats (*Rattus norvegicus*), with the function of attracting females (95); and in male white-tailed deer (*Odocoileus virginianus*), with substantial differences between breeding and non-breeding seasons (96). It was also found in female giant panda (*Ailuropoda melanoleuca*) scent-marks (97), African elephants (*Loxodonta Africana*) musth (98) and wolf (*Canis lupus*) urine with higher levels in males than in females (99). Moreover, this compound was detected in several non-human primate species; namely in the urine of three strepsirrhine primates—Mohol bushbaby (*Galago moholi*), pygmy slow loris (*Nycticebus pygmaeus*) and fat-tailed dwarf lemur (*Cheirogaleus medius*) (100), in the scent gland secretions of owl monkeys (*Aotus nancymaae*) (101), and in both the urine and vaginal odour secretions of Japanese macaques (*Macaca fuscata*) during their fertile period (102). Finally, urinary 2-heptanone increases before menstruation and it is considered to play an important role in the functional changes preceding menstruation in women (103).

3-Heptanone, another aliphatic ketone, was detected in wolf urine (99) and African elephant musth with higher concentrations in older males (98), as well as in urine marks of the aye-aye (*Daubentonia madagascariensis*) (29, 104).

3-Octanone, a dialkyl ketone commonly used as a flavour and fragrance ingredient, has been found across a wide range of mammal species. For instance, it has been detected in the scent odour secretions of the giant panda (97) and in wolf urine (99), while it is involved in signalling musth among male African elephants (98). It was also found in the urine of the aye-aye (100, 105) and in the female anogenital odour secretions of crowned lemurs (*Eulemur coronatus*) (34).

Finally, some major limitations have to be acknowledged for this research work. First of all, the study focused on a small sample of subjects (i.e., one family troop), due to the limited number of breeding pairs hosted by EAZA institutions. Also, due to the seasonality and the constraints related to zoo daily routine, the daily diet and sampling hours varied between the two study periods (breeding vs. non-breeding). Additionally, less vaginal odour samples were collected during the non-breeding period because of the needed change of sampling protocol (i.e., from collection of spontaneously released anogenital scent-marks to training for vaginal odour sampling).

In conclusion, our findings suggest that female anogenital scent-marking and male genital sniffing followed by scent marking may signal the breeding period in gentle lemurs, but neither female nor male behaviours alone can be used as an effective indicator of female ovulation in this lemur species. On the other hand, our chemical investigation supports the hypothesis that anogenital odour may encode information about fertility in female gentle lemurs, with four volatile compounds distinguishing the ovulation window during their breeding period.

Future research work will focus on reproducing the chemical mixture, which may convey information about female fertility, to design, test and implement a new scent enrichment to trigger male mating behaviour and potentially enhance reproductive success in zoo-housed pairs of gentle lemurs.

## Data availability statement

The raw data supporting the conclusions of this article will be made available by the authors, without undue reservation.

## Ethics statement

The animal study was reviewed and approved by the Life Sciences Ethics Committee, University of Wolverhampton (UK) and the Ethics Committee, and Jersey Zoo – Durrell Wildlife Conservation Trust (Channel Islands).

## Author contributions

SF trained the study subjects, conducted the data collection, handling and analyses, and wrote the paper. SV designed the study, conducted the chemical analyses, and assisted with writing the paper. SK conducted the statistical analyses. PA and GM assisted with the endocrinological analyses and the interpretation of the results. All authors contributed to the article and approved the submitted version.

## Funding

This project has received funding from the European Union's Horizon 2020 Research and Innovation Programme under the Marie Skłodowska-Curie grant agreement no. 890341 to SF and SV and from the Primate Society of Great Britain's 2021 Captive Care grant scheme to SF. Lab work and publication fees were funded by the University of Wolverhampton's Research Investment Fund scheme – Phase 4 to SV.

## Conflict of interest

The authors declare that the research was conducted in the absence of any commercial or financial relationships that could be construed as a potential conflict of interest.

## Publisher's note

All claims expressed in this article are solely those of the authors and do not necessarily represent those of their affiliated organizations, or those of the publisher, the editors and the reviewers. Any product that may be evaluated in this article, or claim that may be made by its manufacturer, is not guaranteed or endorsed by the publisher.
